# Social media use and perceptions among pathologists in Turkey: a national survey

**DOI:** 10.1590/1806-9282.20251303

**Published:** 2026-05-01

**Authors:** Busra Ozbek, Seda Duman Ozturk, Semra Uzun Erkal

**Affiliations:** 1 Kocaeli University, School of Medicine, Department of Pathology – Kocaeli, Turkey.

**Keywords:** Social media, Pathology, Surveys and questionnaires, Health personnel, Education

## Abstract

**OBJECTIVE:**

The use of social media is rapidly expanding worldwide. In parallel with the development of digital pathology, the use of digital social media platforms in the field of pathology has also increased. However, as with many other groups, pathologists still have some concerns about using social media. The aim of this study was to investigate the frequency and purposes of social media use among pathologists in their professional and personal lives.

**METHODS:**

This study was conducted using an online survey designed via Google Forms and distributed among pathologists. The questionnaire collected data on participant demographics, the frequency and purposes of social media use, and its application in pathology education and practice.

**RESULTS:**

A total of 124 pathologists participated in the study. All participants reported using social media in their daily lives. Among them, 101 (81.5%) indicated that they also used social media for pathology-related purposes. The platform considered most useful for pathology practice was X (formerly Twitter) (35.6%). Additionally, 98% of participants believed that social media could be beneficial for pathology education.

**CONCLUSION:**

Despite potential sampling bias, our study indicates that among digitally connected pathologists in Turkey, social media, particularly X, is widely adopted as a valuable tool for professional education and practice.

## INTRODUCTION

Social media is increasingly being used across many areas of life. While it is commonly utilized for personal purposes, its adoption in the healthcare sector—particularly among health professionals—has grown rapidly^1^. Social media is widely used by healthcare professionals across various fields for professional purposes and is also extensively utilized by pathologists in both their professional and personal lives^2^. For pathologists new to social media or seeking more in-depth information, various online guides provide valuable insights^3^.

Located at the intersection of Asia and Europe, Turkey embodies distinctive characteristics of both continents. Reflecting influences from Asian, European, and Middle Eastern cultures, and hosting more than fifty ethnic groups, it represents a valuable example in terms of the coexistence and representation of diverse ethnic, racial, and cultural identities^4^. In the context of analyzing the increasingly prevalent use of social media, Turkey occupies a geographically and culturally significant position capable of reflecting a wide spectrum of cultural perspectives. Additionally, Turkey constitutes a particularly relevant example for examining social media usage patterns in developing countries. Turkey's exceptionally high levels of digital connectivity and social media engagement make it a compelling setting for examining professional social-media behaviors among pathologists. For example, recent research found that Turkish internet users increasingly seek health-related information online, with use of social media platforms being a significant component of that behavior^5^. Other studies show that physicians and other healthcare professionals in Turkey are actively engaging in social-media-mediated communication and education^6^. Together, these findings suggest that Turkey offers a rich and meaningful context for investigating how pathologists interact with social media in their professional practice.

Previous studies have shown that pathologists use social media for a range of purposes, including job searching and case consultation^2^. Several studies have investigated the prevalence of social media use among pathologists and the platforms they commonly utilize^7^. To date, no studies have been identified that specifically investigate social media use among pathologists within a defined national context. This study aims to address this gap by examining the prevalence, preferred platforms, and purposes of social media use among pathologists.

## METHODS

A snowball sampling strategy was employed to recruit participants via social media and email. The survey was designed using Google Forms. A preliminary pilot review was conducted with a small group consisting of two pathology residents, two pathology faculty members, and one non-pathology academic to assess face and content validity. The reviewers provided qualitative feedback on the clarity and relevance of the items, as well as on minor grammatical or formatting issues. Revisions were made accordingly prior to dissemination. Informed consent was obtained digitally, with participants indicating their agreement by checking a box on the first page of the survey. Responses were collected between June and August 2023. The survey link was distributed via email, closed Facebook pathology groups, and WhatsApp pathology communities in Turkey. There were no time restrictions for participation, and responses were collected anonymously from volunteers.

The survey was distributed via digital and social media platforms and covered demographic and professional aspects. The first section collected sociodemographic data, including age, gender, current position, institution, years of professional experience, and academic titles where applicable. Subsequent sections explored participants’ use of social media in general and for pathology-specific purposes. These included questions about usage frequency, purposes for using social media in a professional context, preferred platforms, and perceived contributions of social media to pathology education and practice.

All statistical analyses were performed using Jamovi Version 2.6.26.0. The relationships between categorical variables were assessed using the Pearson chi-square test. Fisher's Exact test was used in cases where the expected count in any cell of the contingency table was less than 5. For post-hoc analysis following a significant chi-square result, pairwise comparisons were conducted with a Bonferroni correction applied to the p-value. A p-value of less than 0.05 was considered statistically significant. For the Bonferroni-corrected post-hoc tests, the significance level was adjusted based on the number of comparisons.

## RESULTS

A total of 124 pathologists participated in the study. Sociodemographic characteristics are presented in Table 1. All participants in the survey indicated that they use social media in their daily lives. However, 101 out of 124 participants (81.5%) reported using social media for pathology-related purposes as well. Among the 23 respondents who reported not using social media in pathology practice, 9 (39%) were academics—5 professors, 2 associate professors, and 2 assistant professors. The remaining respondents consisted of six residents (26.1%), five specialists (21.7%), and one each (4.4%) who were a private laboratory manager, a subspecialty resident, and a retired pathologist.

**Table 1:** The sociodemographic data of the participants.

Characteristics		Percentage
Gender (%)	Female	81.5
	Male	17.7
	Prefer not to say	0.8
Age (%)	20–30	14.5
	31–40	38.7
	41–50	21.8
	>50	25
Job title (%)	Specialist	43.6
	Academician	37.1
	Resident	15.3
	Retired	2.4
	Private laboratory manager	0.8
	Cytopathology fellow	0.8
Academic level of academicians (%)	Professor	50
	Associate professor	28.3
	Assistant professor	21.7
Experience (%)	≤5 years	53.7
	>5 years	46.3

Specialist pathologists were the group most frequently using social media for pathology practice, with a usage rate of 81%. Among pathology residents, 31.6% reported not using social media for professional purposes, while this rate was 19.5% among academics. Among retired pathologists, 66.6% reported using social media for pathology-related purposes.

**Figure 1:**
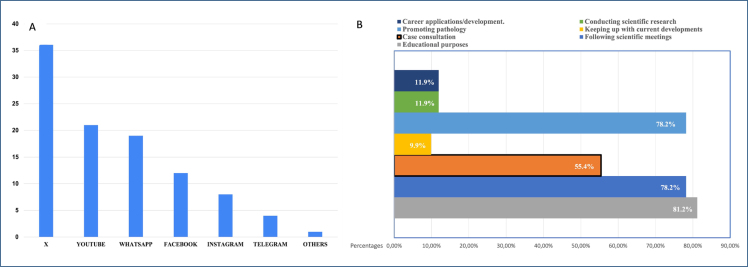
Professional social media preferences among pathologists. **(A)** Platforms considered most useful for pathology practice. **(B)** Types of pathology-related content found most engaging by participants.

When asked about their general social media preferences, 42 participants (41.6%) identified WhatsApp as their most frequently used platform. This was followed by Instagram (18.8%), X (formerly Twitter) (17.8%), YouTube (9.9%), Facebook (7.9%), and both LinkedIn and Telegram (2% each). The most useful platforms are shown in Figure 1A. When platform preferences were analyzed by subgroup, no statistically significant association was found between the professional roles of the participants and their general social media preferences, their platform of choice for pathology-related use, or their use of social media for professional purposes.

When platform preferences were analyzed by subgroup among academics, WhatsApp was the most preferred platform for daily use (48.6%), while X (35.1%) was the most commonly used for pathology practice. Among pathology residents, WhatsApp, X, and Instagram were equally preferred for daily use (30.8% each), whereas X (46.2%) was the most preferred platform for pathology-related use. Among specialists, WhatsApp (36.7%) was the most frequently used for daily purposes, while X (34.7%) was the most commonly used for pathology practice. Retired pathologists who reported using social media indicated a preference for WhatsApp for both daily communication and pathology-related purposes. The most engaging pathology-related content on social media is depicted in Figure 1B.

When evaluating by subgroups, the most engaging content for academics using social media platforms is announcements for scientific meetings/courses (35.1%), followed by case studies (27%). Among pathology residents, tips and tricks capture the most interest (61.5%), with case studies being the second most engaging content at 38.5%. For pathology specialists, tips and tricks are the most engaging content at 47%, followed by case studies at 30.6%.

A significant majority (98%) of survey participants believe that social media can be beneficial for pathology education. A statistically significant relationship was identified between age groups and the social media platform considered most useful for pathology practice (p=0.034). A post-hoc analysis using Bonferroni correction was performed to identify which specific groups differed. With the significance level set at p<0.008, a significant difference was observed between the 20–30 age group and the >50 age group in terms of their platform preferences (p=0.006).

## DISCUSSION AND CONCLUSION

A total of 124 pathologists from Turkey participated in our study, which represents a relatively limited sample size. To date, no studies have specifically examined the use of social media among pathologists in Turkey. Most comparable studies in the literature have been conducted with international participation. Although several of these studies were conducted on an international scale, the absence of detailed information regarding the country-specific distribution of participants makes it challenging to interpret the findings within the unique healthcare, cultural, and systemic contexts of individual nations^7^. Even in studies where country-specific distribution is reported, participation rates have not reached the level observed in our study—even in countries with significantly larger populations, such as the United States^2^. Our study achieved a relatively high participation rate among pathologists who are active on social media platforms; however, this should not be interpreted as representative of all pathologists in Turkey.

As early as 2010, Dr. Michael L. Glassy highlighted that pathologists had begun actively integrating social media into their professional activities, with many contributing to online platforms and blogs dedicated to pathology^8^. However, some physicians expressed negative reactions to this perspective on social media at the time^9^. Even today, some pathologists continue to view the use of social media for professional purposes negatively. Although all participants in our study reported using social media in their personal lives, 81.5% stated that they also use it professionally.

Given that the survey was distributed via Google Forms and primarily promoted through social media channels, it is not unexpected that most participants reported some level of social media use. A significant limitation of this study is the use of a snowball sampling strategy with distribution primarily through social media. This recruitment method introduces a selection bias, as it reaches pathologists who are already digitally active. Consequently, our finding that 81.5% of respondents use social media for professional purposes may not reflect the true proportion in the pathology community in Turkey. However, similar methodological approaches have been successfully employed in previous studies to survey medical professionals^7,10^. Future research should aim to mitigate this bias by employing a stratified sampling method that represents different levels of digital engagement, such as distributing physical surveys at national conferences.

In our findings, specialist pathologists were the group most frequently using social media for pathology practice. Although, specialist pathologists constituted the group most frequently using social media for professional purposes, notable subgroup differences were observed. For instance, 31.6% of residents reported not using social media for pathology practice—the highest proportion among all professional groups—compared with 19.5% of academics. While these differences did not reach statistical significance, they raise questions about potential underlying factors. One possibility is that residents may rely more heavily on direct access to academic supervisors for support, reducing their perceived need for online engagement, whereas some academics may prefer traditional, peer-reviewed resources or may have concerns about data privacy when using social media professionally. As our study focused primarily on usage patterns rather than motivations, further research is needed to explore the reasons behind these subgroup preferences. Future studies could use our findings to investigate the underlying factors influencing professional social media use among pathologists.

In terms of daily social media use, WhatsApp was the most frequently preferred platform among participants (41.6%), followed by Instagram (18.8%) and X (17.8%). According to publicly available online data, Instagram is the most widely used social media platform among the general population in Turkey, suggesting some divergence between general population trends and platform preferences among pathologists^11^. However, in our study, WhatsApp slightly surpassed Instagram as the most frequently used platform among pathologists in Turkey.

For pathology-related purposes, X was the most preferred platform, with 35.6% of participants indicating it as their primary choice. These results align with the previous international studies^7^. The study conducted by Oltulu et al., which included pathologists from multiple countries and utilized two different platforms for survey distribution, differs from our study in terms of both participant demographics and methodology^7^. However, even in that study, X was the most preferred platform among pathologists who participated via X, and it was also preferred for professional purposes by approximately 43% of those who responded through Facebook, matching the preference rate for Facebook itself. These findings are similar to those in our study. However, contrast with results in other medical specialties, highlighting that platform preferences can be highly specific^12^. A study focusing on healthcare professionals, primarily cardiologists, showed that LinkedIn was more commonly preferred for professional purposes, although the most preferred platforms may vary across countries and medical specialties^12^. Another study on radiologists found that LinkedIn was the most preferred platform for professional use among European radiologists, whereas X was more commonly preferred by those in the United States^10^. These findings suggest that preferences for social media platforms may vary not only across countries but also among healthcare professionals working in different specialties. To better frame our results, we have summarized key findings from the literature in comparison with findings of the present study in Table 2. In our study, both academics and specialist pathologists reported a similar preference for WhatsApp for general use, while X was more frequently preferred for pathology-related activities. Among pathology residents, WhatsApp, X, and Instagram were equally preferred for general use.

**Table 2:** Comparison of preferred social media platforms for professional use across various studies.

Study (year)	Specialty	Geographic region	Most preferred platform(s) for professional use	Key findings
This study (2024)	Pathology	Turkey	X	Social media—particularly X—is widely used among pathologists in Turkey for professional purposes.
Oltulu et al. (2018)	Pathology	International	X and Facebook	Pathologists around the world use Twitter and Facebook to enhance professional collaboration and access to educational resources.
Ranschaert et al. (2016)	Radiology	Europe/US	LinkedIn (Europe), X (US)	Facebook is predominantly used for general purposes, whereas LinkedIn and Twitter are preferred for professional engagement.
Guerra et al. (2022)	Cardiology	International	LinkedIn	LinkedIn was the leading platform for professional networking among cardiologists, though its use varied across countries.

US: United States.

The present findings also reflect characteristics unique to the Turkish context. Turkey ranks among the countries with the high levels of social media engagement worldwide, and its healthcare system has undergone rapid digital transformation through national initiatives such as the e-Nabız personal health record platform^13^. Moreover, interpersonal communication and collegial consultation occupy a central role in Turkish medical culture, which may facilitate the use of informal and mobile-based communication tools such as WhatsApp and X for educational and consultative purposes. These sociocultural and technological factors may partly explain the predominance of these platforms among Turkish pathologists. In contrast, previous studies from Western contexts have reported greater professional use of platforms such as LinkedIn and ResearchGate for networking and research dissemination among healthcare professionals^14,15^.

As emphasized in the study by Chen et al., social media offers numerous valuable applications within the field of pathology, supporting education, collaboration, and professional development^16^. Folaranmi and colleagues noted that pathologists in their country used social media for education, case consultation, research collaboration, and professional networking. They emphasized that pathologists in lower-income countries with limited resources could particularly benefit from the opportunities social media provides^17^. In our study, pathologists who used social media reported that they primarily used it for educational purposes and to follow scientific meetings. However, social media was also widely used for case consultations, promoting pathology, and even career-related activities such as job applications and professional development.

Among the various types of content shared on social media, posts featuring tips and tricks were reported as the most engaging overall. However, academic participants showed greater interest in announcements of scientific meetings and educational courses. Similarly, studies in the literature have emphasized the popularity of social media posts related to scientific meetings, particularly those shared during the events themselves^18^. Scientific meetings and conferences have increasingly embraced technological advancements, especially since the COVID-19 pandemic, which led to the rapid expansion of online and hybrid formats. As emphasized in the literature, such formats are essential for providing access to information for individuals who are unable to attend in person^19,20^. Beyond online and hybrid meetings, social media platforms that facilitate online information sharing can significantly enhance pathology education.

Among the pathologists in our study, 98% believed that social media can be beneficial for pathology education. This finding underscores the importance of effectively integrating social media into scientific meetings and educational activities. However, it is important to recognize that this result reflects the participants’ positive perception, not a direct measure of educational efficacy. Such self-reported data may be influenced by factors like social desirability bias. While our results align with studies such as that by Gonzalez and colleagues, which highlighted the educational value of Facebook groups in supporting pathology teaching and learning, they primarily demonstrate a readiness for these tools rather than proven learning outcomes^21^. Therefore, a limitation of our work is its reliance on subjective beliefs. Future studies should move beyond surveys to validate these perceptions, employing more rigorous experimental designs—such as pre/post-test knowledge assessments—to objectively measure the tangible impact of social media on pathology education.

## ETHICAL AND LEGAL CONSIDERATIONS

Our study demonstrates that social media serves as a professional tool that contributes meaningfully to education, consultation, and networking among pathologists in Turkey. Nevertheless, further efforts are needed to enhance awareness and broaden the scope of its professional use. Given that healthcare data are inherently sensitive and require careful handling, the Turkish Ministry of Health enforces strict regulations to maintain patient confidentiality^22^. Pathologists who choose not to engage with social media in their professional practice may do so out of concern for potential ethical or legal implications. However, as the primary objective of our study was to assess usage patterns and frequency among pathologists, data regarding the reasons for non-use were not collected. Future studies addressing the underlying motivations and ethical considerations surrounding social media use may provide a clearer understanding of these factors.

Although all participants in our study reported using social media in their personal lives, 81.5% stated that they also use it professionally. Interestingly, approximately one in five academic pathologists also preferred not to use social media for professional practice. This could reflect a general preference for traditional, peer-reviewed sources, or greater caution regarding public sharing of professional content.

In our findings, specialist pathologists were the group most frequently using social media for pathology practice. Residents and academics, by contrast, reported lower rates of professional use. Despite representing a younger and generally more tech-savvy population, the lower rate of social media use among residents is a surprising finding. One explanation may be that residents rely more on in-house academic supervision, while specialists, required to make independent decisions, may seek broader input via social media. Additionally, residents and academics may be more aware of potential reputational or legal risks, thus adopting a more conservative approach.

Artificial intelligence (AI) technologies are now widely employed in pathology across the continuum—from diagnosis to prognostication—and there is emerging literature indicating the potential use of **large language models** (LLMs) for educational purposes in pathology^23–25^. However, the increasing integration of AI and machine learning into digital pathology introduces new ethical challenges. Although it is currently not possible to identify individual patients from microscopic images, recent studies suggest that advanced AI and machine learning algorithms may be able to extract sensitive personal information from H&E-stained slide images^26^. Even though such technologies remain experimental, these findings raise concerns about the long-term safety of image sharing, even when anonymized.

While sharing anonymized microscopic images does not currently pose legal issues, it is critical to avoid disseminating any personal information that could potentially identify a patient. Although we did not include specific questions in our survey regarding participants’ awareness of ethical risks, these concerns may partially explain the variability in platform engagement among subgroups. Future research should assess how risk perception influences social media behavior and whether targeted guidance or training might increase confidence and responsible use among pathologists.

In conclusion, our study highlights the widespread professional use of social media among pathologists in Turkey, demonstrating its value for education, collaboration, and case consultation. Despite these benefits, further efforts are needed to increase awareness, promote responsible use, and optimize the integration of social media into pathology practice. To support this process, the development of national, specialty-specific guidelines for the ethical and effective use of social media in pathology may help standardize practices and ensure safe, professional engagement across the field.

## Data Availability

The datasets generated and/or analyzed during the current study are available from the corresponding author upon reasonable request.
